# Studies on the Changes of Fermentation Metabolites and the Protective Effect of Fermented Edible Grass on Stress Injury Induced by Acetaminophen in HepG2 Cells

**DOI:** 10.3390/foods13030470

**Published:** 2024-02-02

**Authors:** Tao He, Xianxiu Li, Zhenzhen Wang, Jianwei Mao, Yangchen Mao, Ruyi Sha

**Affiliations:** 1 School of Biological and Chemical Engineering, Zhejiang University of Science and Technology, Hangzhou 310023, China; 2 Zhejiang Provincial Key Laboratory for Chemical & Biological Processing Technology of Farm Product, Hangzhou 310023, China; 3 School of Medicine, University of Southampton, Southampton SO17 1BJ, UK

**Keywords:** edible grass, fermentation, metabolomics, HepG2 cells, acetaminophen-induced stress injury

## Abstract

In this study, gas chromatography-mass spectrometry (GC-MS) based untargeted metabolomics was used to describe the changes of metabolites in edible grass with *Lactobacillus plantarum* (Lp) fermentation durations of 0 and 7 days, and subsequently to investigate the protective effect of fermented edible grass on acetaminophen-induced stress injury in HepG2 cells. Results showed that 53 differential metabolites were identified, including 31 significantly increased and 22 significantly decreased metabolites in fermented edible grass. Fermented edible grass protected HepG2 cells against acetaminophen-induced stress injury, which profited from the reduction in lactate dehydrogenase (LDH) and malondialdehyde (MDA) levels and the enhancement in superoxide dismutase (SOD) activity. Cell metabolomics analysis revealed that a total of 13 intracellular and 20 extracellular differential metabolites were detected. Fermented edible grass could regulate multiple cell metabolic pathways to exhibit protective effects on HepG2 cells. These findings provided theoretical guidance for the formation and regulation of bioactive metabolites in fermented edible grass and preliminarily confirmed the protective effects of fermented edible grass on drug-induced liver damage.

## 1. Introduction

Edible grass is a new food resource obtained by crossing Rumex K-1 (*Rumex patientia* L. × *Rumex tianschanicuscv*) with *Rumex patientia* L. [1]. Recently, much attention has been focused on edible grass because of its characteristics of high yield and high nutritional value. Previous studies have found that edible grass possessed a variety of bioactive compounds, among which phenols, flavonoids and superoxide dismutase (SOD) were closely related to antioxidant activity and hypoglycemic activity [2]. However, as an anti-nutritional component, oxalic acid presented a high level in edible grass [3], which limited the utilization of edible grass in the food field.

Fermentation is a commonly used biotechnology method for food processing and preservation. Apart from prolonging food storage time and reducing antinutritional compounds, fermentation can impart new sensory traits and improve the health benefits of food products [4]. Lactic acid bacteria (LAB) were used for the fermentation of edible grass, and the results have shown that the content of oxalic acid in fermented edible grass was lower than that in unfermented edible grass, whereas the soluble protein content and SOD activity increased significantly during the fermentation process [1]. Not only that, fermented edible grass exhibited stronger scavenging ability of free radicals than unfermented edible grass, and it was confirmed to be effective in protecting HepG2 cells against H_2_O_2_-induced oxidative stress injury [3]. Fermented edible grass has been fully proven to possess antioxidant activity based on prior work, but little research has been done on its other biological activities.

Metabolomics has been widely used for qualitative and quantitative analysis of metabolites and for understanding the transformation relationship between metabolites during the fermentation process [5]. Among various high-throughput analytical techniques, gas chromatography-mass spectrometry (GC-MS) has been used extensively due to its reproducibility, sensitivity and resolution [6]. GC-MS-based metabolomics methods were employed in prior studies to investigate the dynamic changes of metabolites in various fermented foods such as Kimchi [7], paocai [8] and Chinese Huangjiu [9]. In addition, metabolite changes in edible grass during spontaneous fermentation were monitored by GC-MS based metabolomics, and the relationships between the metabolites and antioxidant activity were further clarified. However, little is known about the metabolite profiles of edible grass fermented by LAB. GC-MS metabolomics technology can also be applied to explore cellular metabolite changes under stress conditions to study the protective effect of some bioactive compounds on stress damage of cells [10]. Therefore, the effect of fermented edible grass on cell metabolism could be helpful to reveal the mechanisms of its biological activity.

In this study, metabolites in edible grass fermentation by using *Lactobacillus plantarum* (Lp) were analyzed by GC-MS combined with multivariate statistical methods. HepG2 cells treated with acetaminophen (APAP) were used to establish the liver injury model. The protective mechanisms of fermented edible grass on APAP-induced stress injury in HepG2 cells were evaluated by cell metabolomics. The current study would provide reliable evidence for the potential use of fermented edible grass in functional foods.

## 2. Materials and Methods

### 2.1. Materials

Edible grass was obtained from Zhejiang University of Science and Technology (Hangzhou, China). Lp (1 × 10^11^ CFU/g) was purchased from Shandong Junle Biotechnology Co., Ltd. (Weifang, China). Pyridine, APAP and L-2-chlorophenylalanine were purchased from Shanghai Aladdin Biochemical Technology Co., Ltd. (Shanghai, China). Ribitol was purchased from TCI development Co., Ltd. (Shanghai, China). Methanol was purchased from Chengdu Chron Chemicals Co., Ltd. (Chengdu, China). Methoxyamine hydrochloride and *N*,*O*-bis(trimethylsilyl)trifluoroacetamide (BSTFA) (1% trimethylchlorosilane (TMCS)) were purchased from Shanghai Macklin Biochemical Technology Co., Ltd. (Shanghai, China). The assay kits used for determining protein concentration, lactate dehydrogenase (LDH) level, malondialdehyde (MDA) concentration and SOD activity were obtained from Nanjing Jiancheng Bioengineering Institute (Nanjing, China). HepG2 cells were purchased from Procell Life Science & Technology Co., Ltd. (Wuhan, China).

### 2.2. Edible Grass Fermentation

The fresh edible grass was cleaned with deionized water and pasteurized at 65 °C for 30 min. Then the pasteurized edible grass and sterile water (containing 30% sucrose) were homogenized at a ratio of 1:2 using a juicer. The mixture was inoculated with Lp at 35 °C for 7 days for fermentation. The initial concentration of Lp was 1 × 10^9^ CFU/mL. The fermentation broth was collected on the 7th day. The mixture with no Lp inoculation treatment under the same conditions was considered to be the unfermented edible grass and used as a control.

### 2.3. Cell Viability Determination

After HepG2 cells were thawed and cultured for 48 h, they were seeded in 96-well plates (100 µL, 4 × 10^5^ cells/well) and subsequently cultured at 37 °C for 24 h. Then 100 µL of Dulbecco’s modified Eagle medium (DMEM) was added to the cells and incubated at 37 °C for 24 h to obtain the blank control group. Next, 100 µL of the sample with different diluted times were added to the cells and incubated at 37 °C for 24 h to establish the experimental group. After incubation, cell viability was determined by 3-(4,5-dimethylthiazol-2-yl)-2,5-diphenyltetrazolium bromide (MTT) assay [11] and defined as the ratio of absorbance of the experimental group to the blank control group.

### 2.4. Establishment of Acetaminophen-Induced HepG2 Cells Injury Model

After HepG2 cell suspension was added into 96-well plates (100 µL, 4 × 10^5^ cells/well) and incubated at 37 °C for 24 h, the original culture medium was removed. Different doses of APAP were dissolved in the fresh culture medium, and were then added to the cells to incubate at 37 °C for 24 h. Similarly, the fresh culture medium without APAP was added to the cells to prepare the blank control group. Cell viability was measured by the MTT assay, and APAP at half-maximal inhibitory concentration (IC_50_) was selected for the following experiments.

### 2.5. Determination of Biochemical Indicators of Acetaminophen-Induced HepG2 Cells

HepG2 cell suspension was added into 96-well plates (100 µL, 4 × 10^5^ cells/well) and incubated at 37 °C for 24 h. Then the original culture medium was removed and fresh medium was added to the blank control group and model group (100 µL/well). In the same way, equal volume of silibinin, fermented edible grass and unfermented edible grass dissolved in the fresh medium were added to the positive control group, fermented edible grass experimental group and unfermented edible grass experimental group, respectively. The medium was discarded after 24 h of incubation and APAP at IC_50_ concentration was added to each group except to the blank control group (in which only fresh medium was added) and incubated at 37 °C for 24 h. Cell viability was measured by the MTT assay. Protein concentration was measured using the bicinchoninic acid (BCA) protein concentration assay kit. The MDA concentration, LDH level and SOD activity in HepG2 cells were measured according to the manufacturers’ instructions for the MDA, LDH and SOD assay kits, respectively.

### 2.6. Sample Preparation

A volume of 100 µL of edible grass or fermented edible grass samples, 300 µL of methanol and 5 µL of ribitol (2 mg/mL) were mixed in a vortex instrument for 30 s. Then the mixture was treated with an ultrasonic wave device (ultrasonic power = 400 W) at 0 °C for 10 min, and subsequently centrifuged at 10,000 rpm for 15 min to obtain the supernatant. The samples were successively dried with nitrogen, incubated with 100 μL of methoxyamine hydrochloride (20 mg/mL, dissolved in pyridine) at 80 °C for 30 min, and incubated with 100 μL BSTFA (1% TMCS) at 70 °C for 1.5 h. When all the mixtures were cooled to room temperature, they were centrifuged at 12,000 rpm for 10 min to collect the supernatant for conducting the GC-MS experiment.

According to the results from the MTT experiments, the appropriate diluted time of fermented edible grass was found to be 80 times. HepG2 cells were treated with fermented edible grass followed by APAP according to the method of 2.5 to obtain the fermented edible grass protective group. Moreover, the blank control group and model group were established as described in Section 2.5. The extracellular metabolites were extracted by vortexing 200 μL of cell supernatant medium, 200 μL of methanol, and 20 μL of L-2-chlorophenylalanine (20 mg/mL) for 30 s. Then the mixture was centrifuged at 10,000 rpm at 4 °C for 15 min to obtain the supernatant. The intracellular metabolites were prepared by the following steps. At first, cells were washed three times with PBS and then mixed with 1 mL of methanol/isopropanol solution (3:1, *v*/*v*). Cell collection was conducted by detaching from the culture dishes with a cell scraper, vortexing for 30 s, and ultrasonic treatment at 4 °C for 10 min. Then the collected solutions were placed in liquid nitrogen for 5 min, and dissolved at room temperature. The supernatants were collected by centrifugation at 10,000 rpm at 4 °C for 15 min. Both intracellular and extracellular samples were dried in the vacuum concentrator at 37 °C for 4 h, and then incubated with 80 μL of methoxyamine hydrochloride (20 mg/mL, dissolved in pyridine) at 80 °C for 20 min. The mixture was incubated with 100 μL BSTFA (1% TMCS) at 70 °C for 1 h. When all samples were cooled to room temperature, they were centrifuged at 12,000 rpm for 10 min to collect the supernatant for conducting the GC-MS experiment.

### 2.7. Untargeted Metabolomics Analysis

The untargeted metabolomics analysis was conducted with a GC-MS instrument (Agilent, Santa Clara, CA, USA) equipped with HP-5MS capillary column (30 m × 250 µm, 0.25 µm), following the method described in prior work [3]. The injection volume and temperature were 1 µL and 280 °C, respectively. The temperatures of the transmission line and ion source were 280 °C and 250 °C, respectively. Helium with a flow rate of 1.0 mL/min was used for carrier gas. The heating procedure was shown below: the initial temperature was 40 °C and kept for 1 min, then the temperature was increased to 310 °C at a rate of 8 °C/min, and then the temperature was kept for 10 min. The ionization source was used for full scanning electron bombardment (electron energy = 70 eV) in the single ion monitoring scanning mode. The scanning range was from 50 to 500 *m*/*z*. All detected mass spectra were compared with the NIST 14 standard mass spectrometry database to determine the identity of the compounds. All GC-MS experiments were conducted independently at least five times.

### 2.8. Data Processing and Statistical Analysis

SIMCA-P software (Version 14.0, Umetrics, Umea, Sweden) was used to normalize the metabolite data. Principal component analysis (PCA), projections to latent structure-discriminant analysis (PLS-DA) and orthogonal projections to latent structure-discriminant analysis (OPLS-DA) were used to test the data of all samples. Differential metabolites were defined at the variable importance in the projection values (VIP) of the PLS-DA model (VIP > 1) combined with the Student’s *t*-test (*p* < 0.05). The quantification of identified metabolites was conducted according to the percentage of their own peak area to the total peak area. The metabolic pathway enrichment and visualization of differential metabolites were carried out by MetaboAnalysis 5.0 at https://www.metaboanalyst.ca/ (accessed on 18 July 2023). Statistical analysis was carried out using Origin 2022 software (OriginLab, Northampton, MA, USA) and IBM SPSS Statistics software (Version 26, IBM, Armonk, NY, USA).

## 3. Results and Discussion

### 3.1. Metabolomics Analysis in Edible Grass Fermentation

#### 3.1.1. Multivariate Statistical Analysis

Metabolites in the samples were analyzed using untargeted metabolomics via GC-MS. A total of 90 metabolites were identified, mainly including sugars, organic acids, fatty acids, amino acids, and polyols (Table S1).

**Figure 1:**
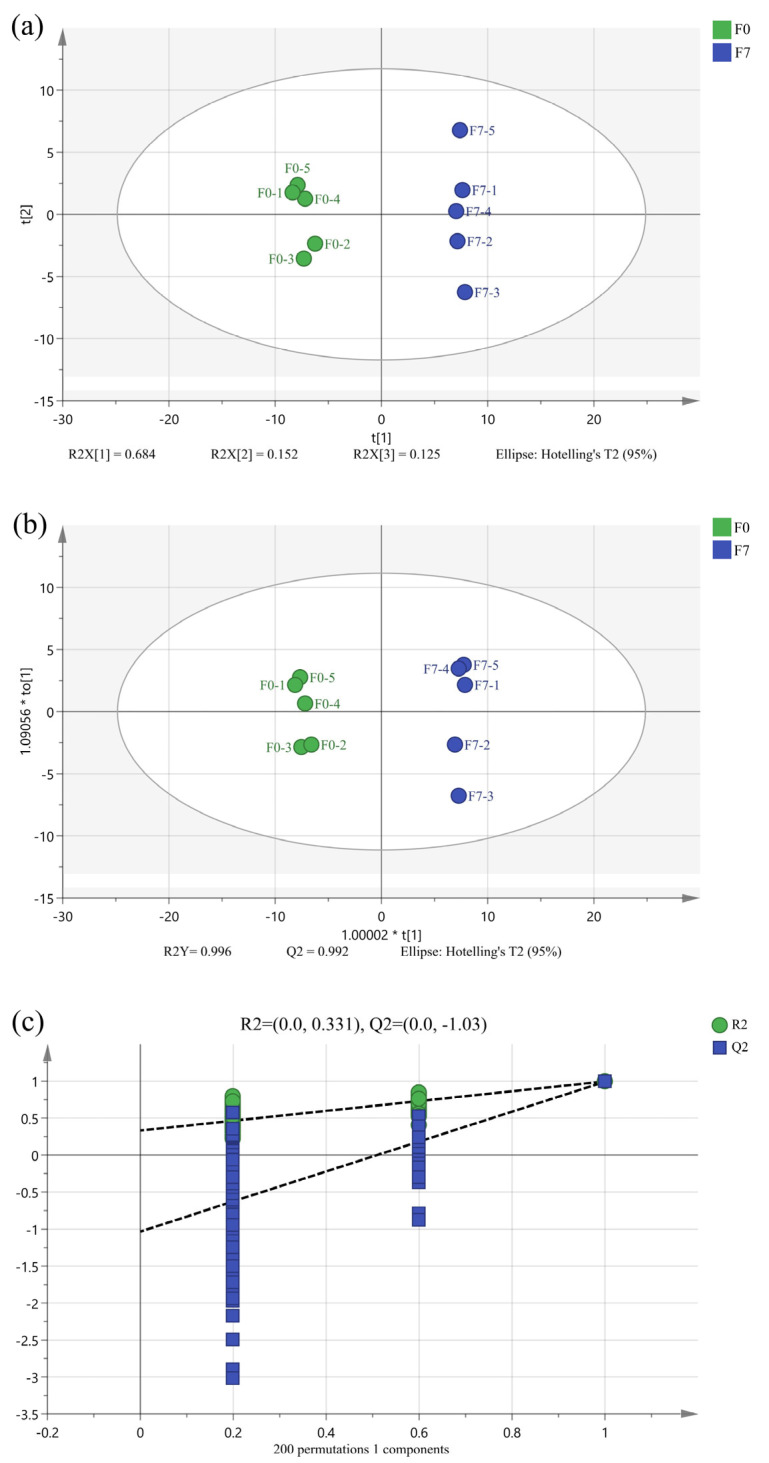
(**a**) PCA score plot, (**b**) OPLS-DA score plot, and (**c**) RPT plot based on the GC-MS data. F0 and F7 represent the unfermented edible grass group and the fermented edible grass group, respectively.

PCA was used to simplify the datasets and acquire important information. As shown in Figure 1a, all samples were included in the ellipse of the confidence level of 95%, indicating that the outlier was non-existent. Moreover, the metabolites on the fermentation time of the 7th day were very different from those on the 0th day, as can be confirmed by the significant separation in the samples before and after fermentation in the PCA score plot. The values of R^2^X and Q^2^ were respectively 0.961 and 0.797, so the PCA model possessed high-fitting and high-predicting properties.

To explore the main differential metabolites in edible grass before and after fermentation, the OPLS-DA model was used to discriminate samples (Figure 1b). The values of R^2^Y and Q^2^ were 0.996 and 0.992, respectively, demonstrating the clear discrimination of this model. Meanwhile, the result (n = 200, Q^2^ = −1.03) of the response permutation testing (RPT) (Figure 1c) verified the stability and reliability of this OPLS-DA model.

#### 3.1.2. Differential Metabolites Analysis

**Figure 2:**
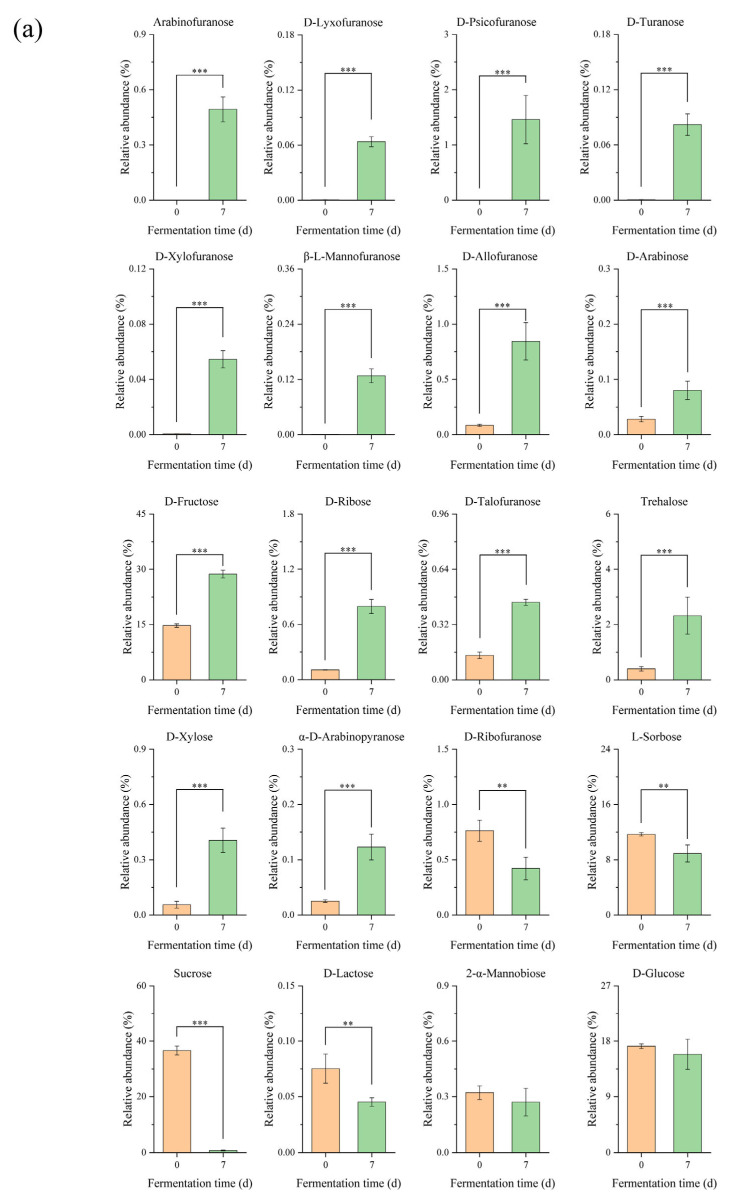

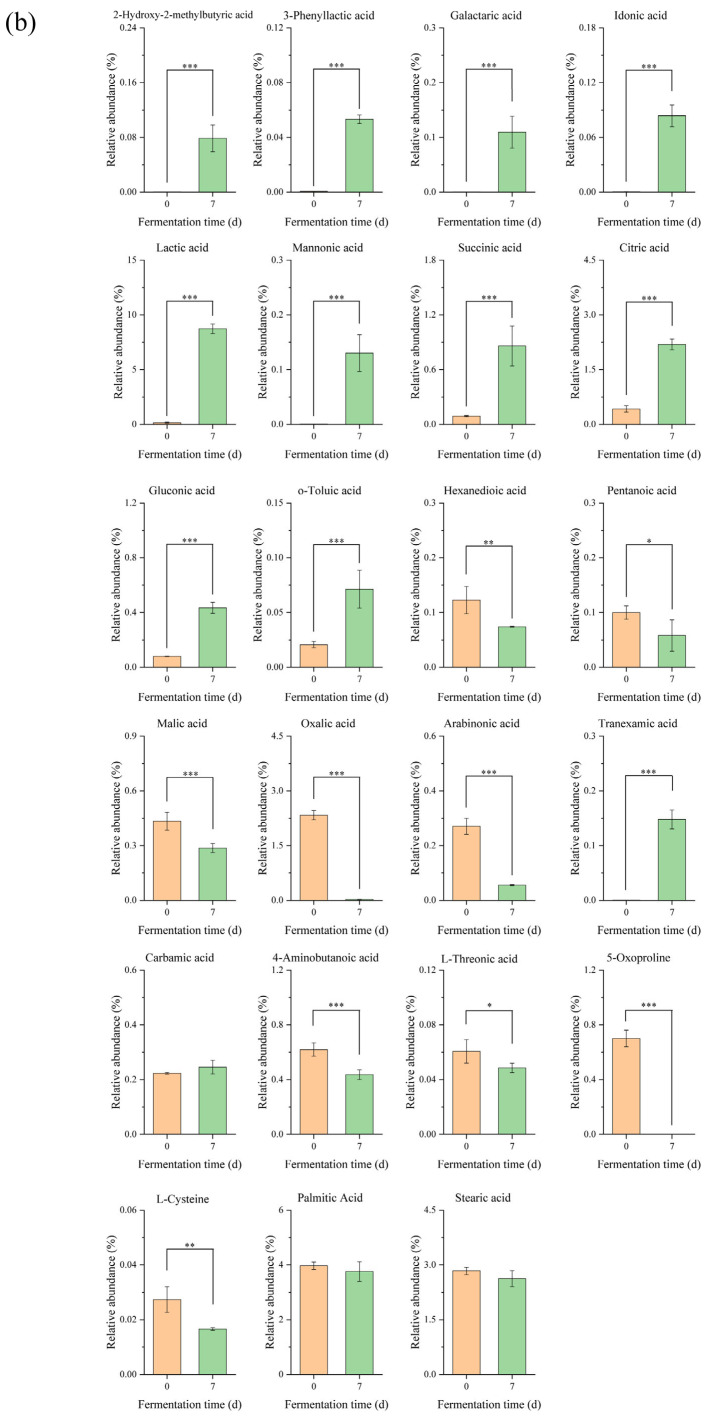

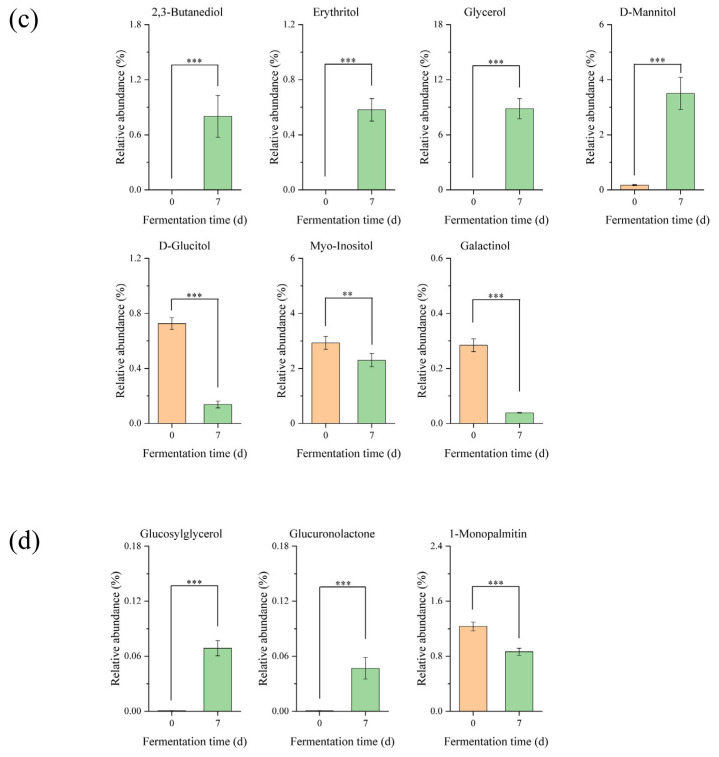
Metabolite changes in (**a**) sugars, (**b**) organic acids, (**c**) polyols, and (**d**) other products of edible grass during fermentation. The samples are as follows: orange color, edible grass; green color, fermented edible grass. “***”, *p* < 0.001, “**”, *p* < 0.01, “*”, *p* < 0.05.

Based on the results of OPLS-DA model analysis, a total of 53 differential metabolites (VIP > 1, *p* < 0.05) were identified in edible grass before and after fermentation. Figure 2 was plotted to understand the changes in differential metabolites before and after fermentation. It was found that the changes in metabolites mainly occurred in sugars and organic acids. During fermentation, sugars provide energy for microbial activities, so the changes in sugars were mainly caused by the metabolic activities of microorganisms [12]. Among all sugar metabolites (Figure 2a), arabinofuranose, D-lyxofuranose, D-turanose, D-psicofuranose, β-L-mannofuranose and D-xylofuranose did not exist in unfermented edible grass, they were produced by fermentation. Generally, oligosaccharides with furan ring structure are the main components of glycopeptides in plant cell walls [13]. For example, arabinogalactan is a component of plant cell walls, and it can be degraded into arabinofuranose and released by the metabolic activity of microorganisms [14,15]. D-Turanose is a kind of sweetener that has α-glucosidase inhibitory activity and anti-inflammatory activity [16], which can be converted from sucrose by amylosucrase [17]. Moreover, the levels of some sugars were low in unfermented edible grass, while increasing significantly due to fermentation. For example, the levels of D-ribose, trehalose and D-xylose in fermented edible grass were 7.4, 5.8 and 7.2 times higher, respectively, than those in unfermented edible grass. D-ribose was reported as a compound with high activity efficacy in relieving symptoms in patients with fibromyalgia and chronic fatigue syndrome [18], and it can be produced from glucose and fructose by fermentation [19]. Trehalose can be obtained from the conversion of glucose by trehalose synthase with the action of microorganisms [20], which was effective on atherosclerosis and postinfarct cardiomyopathy [21,22]. D-xylose played a major role in hypoglycemic activity and antiviral activity [23], and the increase in its level was probably attributed to the release action from hemicellulose by fermentation on edible grass.

Organic acids play a significant role in flavor quality, nutrition value and biological activity of fermented food products [24]. The dissolution of organic acids existed in edible grass and the metabolic activities of microorganisms would lead to changes in organic acid levels. As shown in Figure 2b, most organic acids in fermented edible grass exhibited higher levels than unfermented edible grass. Among these organic acids, lactic acid, succinic acid, citric acid and gluconic acid in fermented edible grass presented at high levels. In the literature, 3-phenyllactic acid has been reported as an antimicrobial compound to enhance the storage features of food products, and it can be produced by the Lp strain [25,26]. Lactic acid was considered as a marker for judging the degree of fermentation, which was produced by the fermentation of lactic acid bacteria [27]. For succinic acid, its level in fermented edible grass was about 9.6 times higher than that in unfermented edible grass. The increase in succinic acid content was considered due to the transformation of α-ketoglutarate in the glyoxylate pathway and tricarboxylic acid (TCA) cycle [28]. Citric acid can be produced by fermentation based on sucrose, and gluconic acid can be produced from glucose by glucose oxidase [29,30]. There are also some organic acids whose relative abundances were decreased after fermentation, such as oxalic acid, arabinonic acid and 5-oxoproline. Oxalic acid usually exists in plants in the form of oxalate and is regarded as an antinutrient compound. Corresponding to the phenomena reported in edible grass fermentation earlier [1,3], the level of oxalic acid in edible grass was significantly reduced after fermentation.

Lactic acid bacteria are a group of microorganisms particularly suited to produce polyols [31]. A total of 7 polyols were detected as differential metabolites (Figure 2c). Compared to edible grass, fermented edible grass displayed a higher level of glycerol and a lower level of myo-inositol, which was similar to the results of kimchi fermentation [32]. The compound, 2,3-butanediol is a promising bulk chemical with wide applications, it can be produced by fermenting a variety of monosaccharides by microorganisms [33]. D-mannitol is a kind of six-carbon diabetic polyol that can be synthesized by fructose [34], so the increase in D-mannitol level was related to the increased fructose level. Both erythritol and glycerol can be obtained by converting glucose in biological production [35,36], so their increased levels benefit from the sufficient supply of glucose.

Glucosylglycerol has the property of low-calorie sweetness and prebiotic properties [36], and glucuronolactone possesses antioxidant, anti-inflammatory and hepatic fibrosis prevention properties [35]. The two compounds were only found in edible grass after fermentation, while the 1-monopalmitin level of fermented edible grass was lower than that of unfermented edible grass (Figure 2d).

### 3.2. Protective Effect of Fermented Edible Grass on Acetaminophen-Induced Liver Injury at the Cellular Level

#### 3.2.1. Effect of Edible Grass, Fermented Edible Grass and Acetaminophen on Cell Viability

No significant difference (*p* > 0.05) was found in the viability of HepG2 cells between the unfermented edible grass experimental group (F0) with sample diluted times of 80, 160 and the blank control group (Figure S1a), and the fermented edible grass experimental group (F7) with sample diluted times of 40, 80 and 160, which also had no side effects on HepG2 cells (Figure S1b). Accordingly, the samples in F0 and F7 were diluted 80 times for subsequent experiments. APAP is considered one of the leading causes of drug-induced liver injury [37]. To construct an APAP-induced HepG2 cell model, the viability of HepG2 cells at different APAP concentrations (10, 13, 20, 40 μmol/L) was investigated, and the results were listed in Figure S1c. It was found that cell viability decreased with the increase of APAP concentration. The IC_50_ value of APAP for HepG2 cells was calculated to be 35.6 μmol/L, so it was chosen for further experiments. Silibinin is a polyphenolic compound that has been used as a hepatoprotective agent for thousands of years [38]. The results showed that the cell viability significantly decreased with the increase in silibinin concentration (*p* < 0.05) (Figure S1d), so 20 µmol/L was selected as the appropriate concentration of silibinin for conducting the follow-up experiments due to its insignificant effect on the inhibition of cell growth.

#### 3.2.2. Protective Effect of Fermented Edible Grass on Acetaminophen-Induced HepG2 Cells

**Figure 3:**
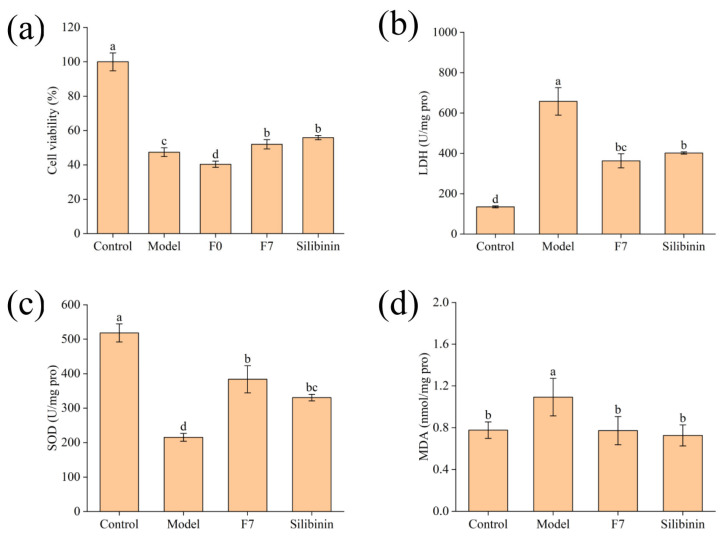
Evaluation of HepG2 cell survival induced by APAP and fermented edible grass protective effects. (**a**) HepG2 cells viabilities, (**b**) LDH level, (**c**) SOD activity, (**d**) MDA level. The bars with different letters indicate significant differences (*p* < 0.05). Control, Model, F0, F7 and Silibinin represent the blank control group, model group, unfermented edible grass group, fermented edible grass group and positive control group, respectively.

As shown in Figure 3a, both fermented edible grass and silibinin could significantly inhibit the cell death induced by APAP as reflected by the higher cell viability, compared to that of the model group (*p* < 0.05). However, unfermented edible grass could not reduce the damage induced by APAP in HepG2 cells. The phenomenon indicated that fermented edible grass played a protective role against APAP-induced stress injury in HepG2 cells.

To further verify the protective effect of fermented edible grass on APAP-induced HepG2 cells, the values of LDH, MDA and SOD activity of HepG2 cells were measured and listed in Figure 3. As shown in Figure 3b, the model group exhibited higher LDH level compared to the control group, and the LDH level in HepG2 cells pretreated with the fermented edible grass and silibinin was significantly lower than that in the model group (*p* < 0.05). After being treated with APAP, the SOD activity in HepG2 cells was decreased by 58% compared with that in the negative control group (Figure 3c). However, when pretreated with the fermented edible grass, the SOD activity in HepG2 cells was significantly higher than that in the model group (*p* < 0.05). As shown in Figure 3d, MDA content in the model group was significantly higher than the negative control group (*p* < 0.05). The content of MDA in F7 was significantly lower (*p* < 0.05) than the model group, and it had no significant difference (*p* > 0.05) compared to the positive control group. The results confirmed that the fermented edible grass could protect HepG2 cells from APAP-induced stress injury due to the enhancement of LDH level and SOD activity and the reduction of MDA content in HepG2 cells.

### 3.3. Analysis of Intracellular and Extracellular Metabolomics in HepG2 Cells

#### 3.3.1. Multivariate Statistical Analysis

**Figure 4:**
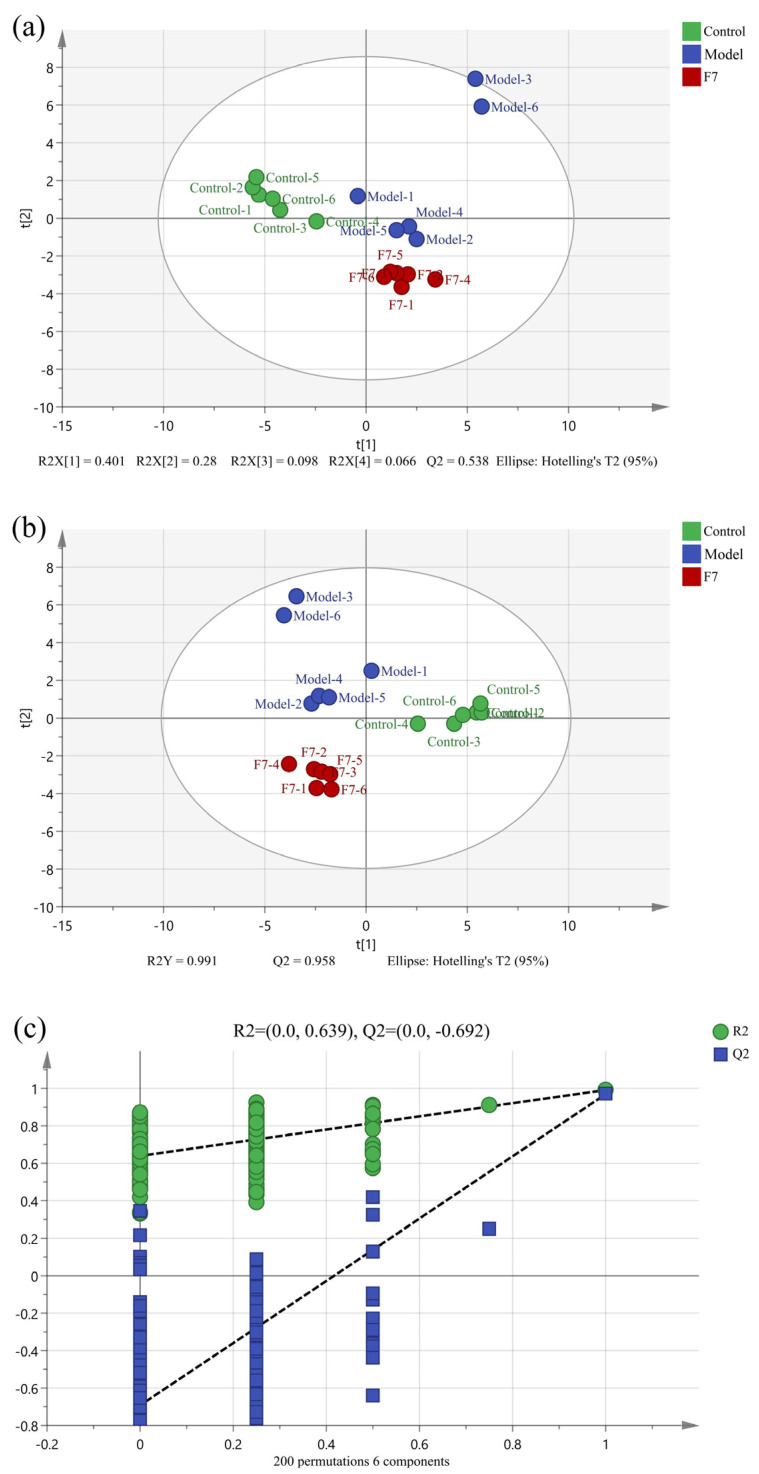
(**a**) PCA score plot, (**b**) PLS-DA score plot, and (**c**) RPT plot based on the GC-MS data of intracellular metabolites. Control, Model and F7 represent the blank control group, model group and fermented edible grass group, respectively.

**Figure 5:**
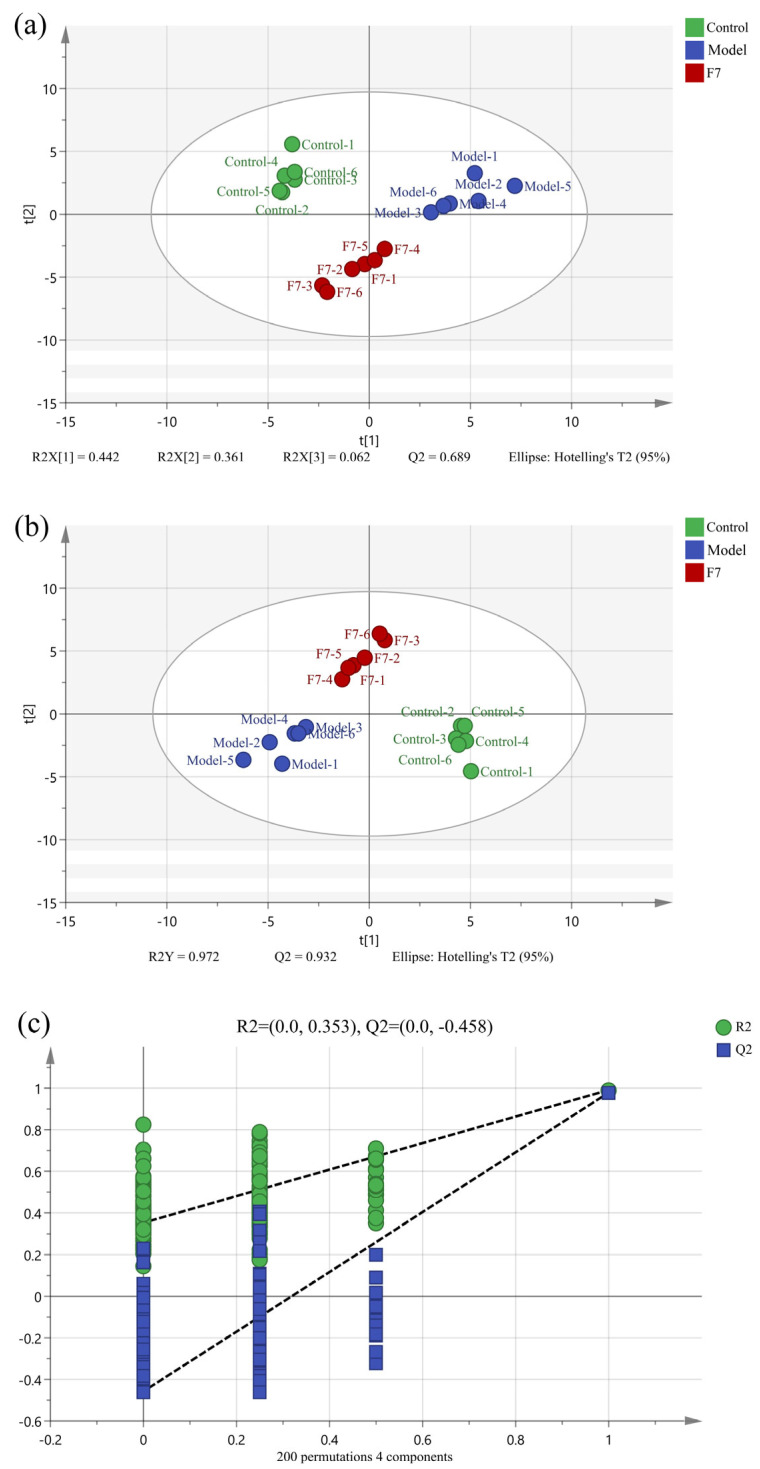
(**a**) PCA score plot, (**b**) PLS-DA score plot, and (**c**) RPT plot based on the GC-MS data of extracellular differential metabolites. Control, Model and F7 represent the blank control group, model group and fermented edible grass group, respectively.

Figure 4a and Figure 5a showed the PCA score plots of the three groups, from which it can be seen that the three groups can be distinguished from each other. To exclude some factors that could affect the accuracy of the results, PLS-DA was used to process the data. As shown in Figure 4b and Figure 5b, the three groups of both intracellular and extracellular samples had a separation tendency, indicating that the treatment of both APAP and fermented edible grass could lead to a significant change in intracellular and extracellular metabolites. The values of R^2^Y (0.991 and 0.972) and Q^2^ (0.958 and 0.932) are close to 1, indicating good fitness of the PLS-DA model. Besides, RPT was used to prove the validity of the model (Figure 4c and Figure 5c). The results showed that the Q^2^ values in intracellular and extracellular sample detection were −0.692 and −0.458, indicating that the PLS-DA model was not overfitting. In conclusion, both cell injury induced by APAP and the protective effects of fermented edible grass led to significant changes in intracellular and extracellular metabolites.

#### 3.3.2. Identification of Differential Metabolites

A total of 32 intracellular metabolites (Table S2) and 31 extracellular metabolites were detected (Table S3). Among them, 13 intracellular differential metabolites and 20 extracellular differential metabolites were screened out according to the screening of metabolites with VIP > 1 and *p* < 0.05 (Table 1 and Table 2).

Among all intracellular differential metabolites (Table 1), compared with the blank control group, the levels of L-phenylalanine, 3-cyclohexene-1-ethanol and propyleneglycol monoleate in the model group were upregulated, while glycine, serine, L-glutamic acid, D-mannose, sucrose, and cholesterol showed a downward trend, and other differential metabolites had no significant change. In the fermented edible grass group, L-valine, succinic acid, D-mannose, L-phenylalanine, gluconic acid, eicosyl isopropyl ether and propyleneglycol monoleate had significant changes compared with those in the model group. Notably, D-mannose, L-phenylalanine and propyleneglycol monoleate in the fermented edible grass group showed a reverse trend compared with those in the model group.

As shown in Table 2, the levels of extracellular propanoic acid, L-valine, L-leucine, L-threonine, glutamine, L-phenylalanine, L-asparagine, 3-methyl-3-pentanol and D-galactose in the model group were increased compared with those in the blank control group, while the levels of urea, L-proline, glycine, serine, n-butylamine, butane, L-ornithine, 1,2,3-propanetricarboxylic acid and D-mannitol were downregulated, and other extracellular differential metabolites had no significant change. Compared with the model group, the levels of propanoic acid, L-valine, L-leucine, L-threonine, butane, glutamine, L-phenylalanine, L-asparagine, 3-Methyl-3-pentanol and D-galactose in the fermented edible grass group showed an opposite trend, while other extracellular differential metabolites exhibited a downward trend or kept unchanged.

#### 3.3.3. Analysis of Metabolic Pathway

The Kyoto Encyclopedia of Genes and Genomes (KEGG) database was used to explore the metabolic pathway enrichment of the differential metabolites. According to impact value > 0.1, the intracellular differential metabolites were mainly enriched in 9 metabolic pathways, including amino acid metabolism and biosynthesis, aminoacyl-tRNA biosynthesis, glyoxylate and dicarboxylate metabolism and glutathione metabolism (Table 3). The key intracellular metabolic pathways involved 6 differential metabolites, namely succinic acid, L-valine, L-phenylalanine, serine, glycine, and L-glutamic acid. Besides, the extracellular differential metabolites were mainly enriched in 9 metabolic pathways, involving amino acid metabolism and biosynthesis, aminoacyl-tRNA biosynthesis, glyoxylate and dicarboxylate metabolism and inositol phosphate metabolism (Table 4). The differential metabolites related to the key extracellular metabolic pathways were D-galactose, L-threonine, inositol, serine, L-proline, glycine, glutamine, L-phenylalanine, L-alanine, L-asparagine, L-ornithine, L-leucine and L-valine, respectively.

Phenylalanine can be transformed into phenylpyruvate by reacting with ketoglutarate, which leads to the accumulation of phenylpyruvate in the body to affect adversely human health [39]. The intracellular and extracellular L-phenylalanine level in the model group was up-regulated compared to the control group, causing damage to the cells. Fermented edible grass treatment reversed the upward trend, and reduced cell damage caused by APAP. L-Valine is an essential amino acid that plays an important role in protein degradation and conversion, glycogen synthesis, energy metabolism, etc. [40]. It was reported that L-valine can protect the liver by supplementing branched-chain amino acids, reducing hepatocyte apoptosis and promoting hepatocyte regeneration [41]. This is consistent with the up-regulation of intracellular L-valine in the fermented edible grass protective group. L-Threonine is an essential amino acid that must be obtained from the diet [42]. Glutamine has many important physiological functions, including regulation of metabolism and immune status and enhancement of antioxidant capacity [39]. L-Asparagine plays an important role in protein synthesis [43]. L-Leucine is an essential amino acid that can stimulate protein synthesis, increase the reutilization of amino acids in many organs, and reduce protein breakdown [44]. Compared with the control group, the levels of extracellular L-threonine, glutamine, L-asparagine, and L-leucine were upregulated after the cells were induced by APAP, indicating that the cell metabolism was inhibited by APAP-induced cell injury. Fermented edible grass treatment reversed the downward trend of the four amino acids and increased utilization of them. L-alanine is an important precursor for energy metabolism and can be converted into some biomolecules [45]. No significant difference was found in the level of extracellular L-alanine between the control group and the model group (*p* > 0.05), while the level of L-alanine in the fermented edible grass group was downregulated compared with the model group, indicating that fermented edible grass could reduce the degree of APAP-induced cell injury by promoting the transport and metabolism of L-alanine. These varying trends revealed that the protective effect of fermented edible grass on APAP-induced cell injury has a close relationship to metabolism of these amino acids.

Succinic acid is able to activate the succinate receptor 1 (SUCNR1 or GPR91) in the liver and facilitate reparative processes in the liver [46]. Compared with the model group, the fermented edible grass group showed an increased level of intracellular succinic acid, indicating that fermented edible grass could reduce cell damage by providing succinic acid. D-galactose can lead to degenerative changes in physiological and functional organs such as heart aging and liver damage [47]. It was found that the level of extracellular D-galactose in the model group was higher than that in the control group, while it was downregulated after fermented edible grass pre-treatment, indicating that the metabolic transformation of D-galactose was promoted by the treatment of fermented edible grass.

## 4. Conclusions

In conclusion, a total of 53 metabolites including sugars, organic acids and polyols were identified as differential metabolites in edible grass before and after fermentation. Fermented edible grass showed its protection for APAP-induced stress injury in HepG2 cells, as reflected by increasing vitality of cells, reducing the levels of LDH and MDA and increasing the activity of SOD. Cell metabolomics results revealed that the protective effect of fermented edible grass on APAP-induced stress injury was mainly due to the regulation of multiple cell metabolic pathways. The comprehensive view of the metabolome variations of edible grass during Lp fermentation will be beneficial for future studies on the enhancement of the nutritional value and biological properties of fermented edible grass, and the cellular metabolomics analysis will provide reliable evidence for the potential use of fermented edible grass in functional foods. The effect of fermented edible grass on APAP-induced HepG2 cells preliminarily confirmed the biological activity of fermented edible grass, while the digestion and absorption of compounds existing in fermented edible grass in the stomach and gut would influence the effectiveness of fermented edible grass. Therefore, future studies are still required to establish the APAP-induced liver injury model in mice to further demonstrate the protective effects of fermented edible grass on drug-induced liver damage.

## Figures and Tables

**Table 1:** Statistics of intracellular differential metabolites.

R.T.	Metabolite	VIP	Trend	
			Mod vs. Con	F7 vs. Mod
11.976	L-Valine	1.35	NC	↑
13.567	Glycine	1.02	↓	NC
14.551	Serine	1.12	↓	NC
17.177	Succinic acid	1.38	NC	↑
18.533	L-Glutamic acid	1.01	↓	NC
22.499	D-Mannose	1.18	↓	↑
22.779	L-Phenylalanine	1.25	↑	↓
23.489	Gluconic acid	1.38	NC	↓
29.354	Eicosyl isopropyl ether	1.08	NC	↓
30.618	Sucrose	1.15	↓	NC
34.715	Cholesterol	1.03	↓	NC
34.944	3-Cyclohexene-1-ethanol	1.14	↑	NC
36.729	Propyleneglycol monoleate	1.33	↑	↓

↑, significant upregulation; ↓, significant downregulation; NC, no significance; Mod, model group; Con, blank control group; F7, fermented edible grass group.

**Table 2:** Statistics of extracellular differential metabolites.

R.T.	Metabolite	VIP	Trend	
			Mod vs. Con	F7 vs. Mod
9.069	Propanoic acid	1.04	↑	↓
9.865	Alanine	1.04	NC	↓
11.976	L-Valine	1.01	↑	↓
12.371	Urea	1.12	↓	↓
12.977	L-Leucine	1.02	↑	↓
13.378	L-Proline	1.10	↓	↓
13.567	Glycine	1.09	↓	NC
14.551	Serine	1.11	↓	↓
14.992	L-Threonine	1.12	↑	↓
16.062	n-Butylamine	1.03	↓	NC
16.994	Butane	1.10	↓	↑
18.528	Glutamine	1.09	↑	↓
18.596	L-Phenylalanine	1.07	↑	↓
19.283	L-Asparagine	1.04	↑	↓
21.263	L-Ornithine	1.03	↓	↓
21.372	1,2,3-Propanetricarboxylic acid	1.05	↓	NC
22.007	3-Methyl-3-pentanol	1.01	↑	↓
22.722	D-Galactose	1.14	↑	↓
22.991	D-Mannitol	1.10	NC	↓
24.770	Myo-inositol	1.11	↓	↓

↑, significant upregulation; ↓, significant downregulation; NC, no significance; Mod, model group; Con, blank control group; F7, fermented edible grass group.

**Table 3:** The metabolic pathway analysis of the intracellular differential metabolites.

No.	Metabolic Pathway Name	Impact ^a^	Hits ^b^
1	D-Glutamine and D-glutamate metabolism	0.500	1
2	Phenylalanine, tyrosine and tryptophan biosynthesis	0.500	1
3	Glycine, serine and threonine metabolism	0.463	2
4	Phenylalanine metabolism	0.357	1
5	Alanine, aspartate and glutamate metabolism	0.197	2
6	Aminoacyl-tRNA biosynthesis	0.167	5
7	Glyoxylate and dicarboxylate metabolism	0.148	3
8	Arginine biosynthesis	0.117	1
9	Glutathione metabolism	0.108	2

^a^ The importance of the metabolic pathway in the whole metabolic pathway network, ^b^ the number of differential metabolites contained in the metabolic pathway.

**Table 4:** The metabolic pathway analysis of the extracellular differential metabolites.

No.	Metabolic Pathway Name	Impact ^a^	Hits ^b^
1	Phenylalanine, tyrosine and tryptophan biosynthesis	0.500	1
2	Glycine, serine and threonine metabolism	0.463	3
3	Phenylalanine metabolism	0.357	1
4	Galactose metabolism	0.356	2
5	Arginine and proline metabolism	0.188	2
6	Aminoacyl-tRNA biosynthesis	0.167	10
7	Glyoxylate and dicarboxylate metabolism	0.148	3
8	Inositol phosphate metabolism	0.129	1
9	Alanine, aspartate and glutamate metabolism	0.114	3

^a^ The importance of the metabolic pathway in the whole metabolic pathway network, ^b^ the number of differential metabolites contained in the metabolic pathway.

## Data Availability

The original contributions presented in the study are included in the article and Supplementary Materials, further inquiries can be directed to the corresponding author.

## Supplementary Materials

The following supporting information can be downloaded at: https://www.mdpi.com/article/10.3390/foods13030470/s1, Figure S1: The viabilities of HepG2 cells treated with (a) unfermented edible grass, (b) fermented edible grass, (c) APAP, and (d) silibinin at different concentrations. Different superscript letters in the columns indicate significant differences (*p* < 0.05); Table S1: Identification of metabolites by GC-MS during fermentation; Table S2: Identification of intracellular metabolites by GC-MS; Table S3: Identification of extracellular metabolites by GC-MS.
