# Supplementary material for: Studies on the Changes of Fermentation Metabolites and the Protective Effect of Fermented Edible Grass on Stress Injury Induced by Acetaminophen in HepG2 Cells
Source: Foods. 2024 Feb 2;13(3):470. doi: 10.3390/foods13030470 (PMC10855311; doi:10.3390/foods13030470)
Supplement: Supplementary file 1 [file foods-13-00470-s001.zip › foods-2832181-supplementary.pdf]

Studies on the Changes of Fermentation Metabolites and the Protection of Fermented Edible Grass from Stress Injury Induced by Acetaminophen in HepG2 Cells

Tao He[1,2], Xianxiu Li[1,2,*], Zhenzhen Wang[1,2], Jianwei Mao[1,2], Yangchen Mao[3], and Ruyi Sha[1,2]

[1]School of Biological and Chemical Engineering, Zhejiang University of Science and Technology, Hangzhou 310023, China
[2]Zhejiang Provincial Key Laboratory for Chemical & Biological Processing Technology of Farm Product, Hangzhou 310023, China
[3]School of Medicine, University of Southampton, Southampton SO17 1BJ, UK

*Correspondence: lixianxiu@zust.edu.cn

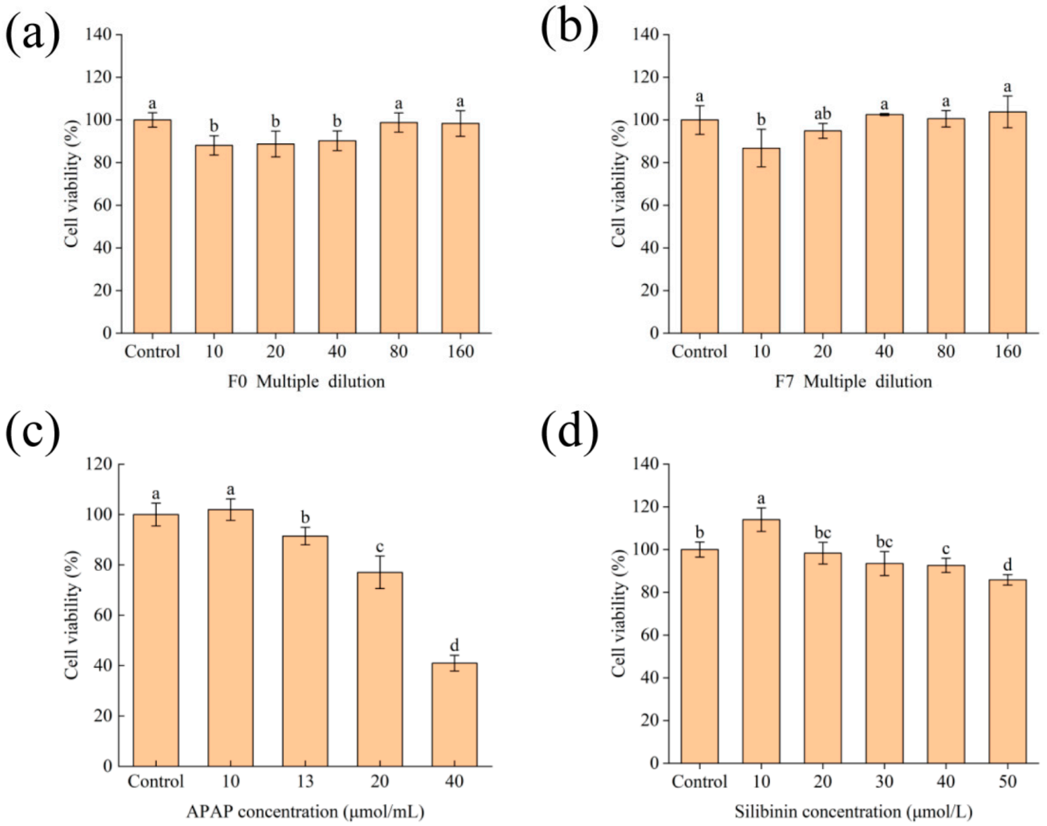

Supplemental Figure S1. The viabilities of HepG2 cells treated with (a) unfermented edible grass, (b) fermented edible grass, (c) APAP, and (d) silibinin at different concentrations. Different superscript letters in the columns indicate significant differences (p<0.05).

Supplemental Table S1. Identification of metabolites by GC-MS during fermentation.

No.	Class	Metabolite identity	Retention time	Molecular formula	VIP
1	Sugar	D-Glucose	22.837	$C_6H_{12}O_6$	1.185
2	Sugar	D-Erythrose	18.366	$C_4H_8O_4$	1.228
3	Sugar	D-Lyxofuranose	18.534	$C_5H_{10}O_5$	1.085
4	Sugar	β-D-Galactofuranose	18.651	$C_6H_{12}O_6$	0.612
5	Sugar	D-Ribofuranose	18.802	$C_5H_{10}O_5$	0.982
6	Sugar	α-D-Arabinopyranose	19.121	$C_5H_{10}O_5$	0.840
7	Sugar	D-Arabinose	19.574	$C_5H_{10}O_5$	0.872
8	Sugar	D-Tagatofuranose	19.692	$C_6H_{12}O_6$	0.769
9	Sugar	D-Ribose	19.792	$C_5H_{10}O_5$	0.994
10	Sugar	Xylopyranose	20.958	$C_5H_{10}O_5$	1.032
11	Sugar	Arabinofuranose	21.143	$C_5H_{10}O_5$	0.994
12	Sugar	D-Fructofuranose	21.52	$C_6H_{12}O_6$	0.755
13	Sugar	D-Xylose	21.805	$C_5H_{10}O_5$	1.018
14	Sugar	D-Fructose	22.46	$C_6H_{12}O_6$	1.294
15	Sugar	L-Sorbose	22.737	$C_6H_{12}O_6$	1.274
16	Sugar	D-Mannose	23.148	$C_6H_{12}O_6$	1.523
17	Sugar	D-Allofuranose	23.466	$C_6H_{12}O_6$	1.032
18	Sugar	β-D-Glucopyranose	23.743	$C_6H_{12}O_6$	0.497
19	Sugar	D-Galactose	25.337	$C_6H_{12}O_6$	0.703
20	Sugar	D-Mannopyranose	27.526	$C_6H_{12}O_6$	1.015
21	Sugar	D-Xylofuranose	29.347	$C_5H_{10}O_5$	0.907
22	Sugar	β-L-Mannofuranose	30.337	$C_6H_{12}O_6$	0.982
23	Sugar	Sucrose	30.865	$C_{12}H_{22}O_{11}$	0.990
24	Sugar	D-Psicofuranose	31.369	$C_6H_{12}O_6$	0.940
25	Sugar	Trehalose	31.822	$C_{12}H_{22}O_{11}$	1.138
26	Sugar	Maltose	32.048	$C_{12}H_{22}O_{11}$	0.664
27	Sugar	D-Talofuranose	32.208	$C_6H_{12}O_6$	1.031
28	Sugar	2-α-Mannobiose	32.291	$C_{12}H_{22}O_{11}$	1.019
29	Sugar	D-Turanose	32.426	$C_{12}H_{22}O_{11}$	0.952
30	Sugar	Palatinose	32.526	$C_{12}H_{22}O_{11}$	0.733
31	Sugar	D-Cellobiose	32.677	$C_{12}H_{22}O_{11}$	0.814
32	Sugar	Lactulose	32.736	$C_{12}H_{22}O_{11}$	1.079
33	Sugar	β-Gentiobiose	32.979	$C_{12}H_{22}O_{11}$	0.861
34	Sugar	D-Lactose	33.063	$C_{12}H_{22}O_{11}$	1.280
35	Sugar	3-α-Mannobiose	33.247	$C_{12}H_{22}O_{11}$	0.921
36	Organic acid	Lactic acid	9.525	$C_3H_6O_3$	1.398
37	Organic acid	Glycolic acid	9.868	$C_2H_4O_3$	0.919
38	Organic acid	Oxalic acid	10.775	$C_2H_2O_4$	1.016
39	Organic acid	2-Hydroxy-2-methylbutyric acid	11.429	$C_5H_{10}O_3$	0.964

40	Organic acid	Boric acid	11.856	BH_3O_3	0.869
41	Organic acid	Propanedioic acid	12.075	$C_3H_4O_4$	0.723
42	Organic acid	Pentanoic acid	12.729	$C_5H_{10}O_2$	0.874
43	Organic acid	Tartronic acid	12.939	$C_3H_4O_5$	0.436
44	Organic acid	Butanedioic acid	13.887	$C_4H_6O_4$	1.225
45	Organic acid	o-Toluic acid	14.692	$C_8H_8O_2$	1.073
46	Organic acid	D-Citramalic acid	16.588	$C_5H_8O_5$	1.140
47	Organic acid	Malic acid	16.831	$C_4H_6O_5$	1.132
48	Organic acid	Hexanedioic acid	16.974	$C_6H_{10}O_4$	0.973
49	Organic acid	Pentanedioic acid	18.123	$C_5H_8O_4$	1.156
50	Organic acid	3-Phenyllactic acid	18.224	$C_9H_{10}O_3$	0.981
51	Organic acid	2,3,4,5-Tetrahydroxyp-entanoic acid	19.021	$C_5H_{10}O_6$	1.593
52	Organic acid	Gluconic acid	19.364	$C_6H_{12}O_7$	0.914
53	Organic acid	Mannonic acid	20.228	$C_6H_{12}O_7$	1.327
54	Organic acid	Citric acid	21.629	$C_6H_8O_7$	1.074
55	Organic acid	D-Gluconic acid	24.062	$C_6H_{12}O_7$	0.678
56	Organic acid	Galactaric acid	24.557	$C_6H_{10}O_8$	0.938
57	Organic acid	α-D-Glucopyranuronic acid	28.13	$C_6H_{10}O_7$	0.842
58	Organic acid	Idonic acid	33.138	$C_6H_{12}O_7$	0.892
59	Organic acid	2-Ethylbutyric acid	34.783	$C_6H_{12}O_2$	0.604
60	Organic acid	Arabinonic acid	37.635	$C_5H_{10}O_6$	1.048
61	Organic acid	Glyceric acid	14.289	$C_3H_6O_4$	0.735
62	Organic acid	L-Threonic acid	18.031	$C_4H_5O_5$	0.829
63	Fatty acid	Arachidic acid	28.374	$C_{20}H_{40}O_2$	0.752
64	Fatty acid	Palmitic Acid	24.146	$C_{16}H_{32}O_2$	0.918
65	Fatty acid	Stearic acid	26.335	$C_{18}H_{36}O_2$	0.936
66	Amino acid	Ethanimidic acid	10.917	C_2H_5NO	0.988
67	Amino acid	L-Cysteine	13.073	$C_3H_7NO_2S$	0.917
68	Amino acid	Glycine	13.132	$C_2H_5NO_2$	0.703
69	Amino acid	L-Threose	15.581	$C_2H_5NO_2$	0.855
70	Amino acid	Sarcosine	17.066	$C_3H_7NO_2$	1.265
71	Amino acid	5-Oxoproline	17.284	$C_5H_7NO_3$	0.996
72	Amino acid	4-Aminobutanoic acid	17.402	$C_4H_9NO_2$	0.978
73	Amino acid	Tranexamic acid	25.882	$C_8H_{15}NO_2$	0.978
74	Amino acid	Carbamic acid	35.655	CH_3NO_2	0.711
75	Polyol	2,3-Butanediol	9.063	$C_4H_{10}O_2$	1.274
76	Polyol	Glycerol	13.35	$C_3H_8O_3$	1.083
77	Polyol	Xylitol	20.446	$C_5H_{12}O_5$	1.030
78	Polyol	D-Glucitol	20.849	$C_6H_{14}O_6$	1.009
79	Polyol	D-Mannitol	23.273	$C_6H_{14}O_6$	1.426
80	Polyol	Myo-Inositol	25.035	$C_6H_{12}O_6$	0.954
81	Polyol	Galactinol	33.935	$C_{12}H_{22}O_{11}$	1.032

82	Polyol	1,2,3-Propatriol	35.538	$C_5H_8O_5$	0.438
83	Polyol	Erythritol	17.234	$C_4H_{10}O_4$	0.998
84	Other	Glucuronolactone	12.377	$C_6H_8O_6$	0.873
85	Other	Ethanolamine	15.866	C_2H_7NO	0.660
86	Other	Glyceryl-glycoside	27.35	$C_9H_{18}O_8$	1.139
87	Other	Methyl galactoside	29.464	$C_7H_{14}O_6$	0.709
88	Other	Glycerol monostearate	31.662	$C_{21}H_{42}O_4$	1.279
89	Other	Glucoheptonolactone	31.964	$C_7H_{12}O_7$	1.425
90	Other	1-Monopalmitin	29.901	$C_{19}H_{38}O_4$	1.030

Supplemental Table S2. Identification of intracellular metabolites by GC-MS.

No.	Class	Metabolite identity	Retention time	Molecular formula	VIP
1	Sugar	D-Xylose	22.178	$C_5H_{10}O_5$	0.873
2	Sugar	D-Fructose	22.310	$C_6H_{12}O_6$	0.571
3	Sugar	D-Mannose	22.499	$C_6H_{12}O_6$	0.904
4	Sugar	Sucrose	30.618	$C_{12}H_{22}O_{11}$	0.817
5	Organic acid	Lactic Acid	9.069	$C_3H_6O_3$	1.012
6	Organic aced	Propanoic acid	10.597	$C_3H_6O_2$	0.850
7	Organic aced	1,2-Butanediol	11.244	$C_4H_{10}O_2$	0.522
8	Organic aced	Succinic acid	17.177	$C_4H_6O_4$	1.179
9	Organic aced	Gluconic acid	23.489	$C_6H_{12}O_7$	1.248
10	Organic aced	acetic acid	24.004	$C_2H_4O_2$	1.385
11	Fatty acid	Palmitic Acid	23.895	$C_{16}H_{32}O_2$	0.872
12	Fatty acid	Stearic acid	26.121	$C_{18}H_{36}O_2$	0.897
13	Amino acid	L-Alanine	9.870	$C_3H_7NO_2$	0.861
14	Amino acid	L-Valine	11.976	$C_5H_{11}NO_2$	0.924
15	Amino acid	L-Norleucine	12.977	$C_6H_{13}NO_2$	1.083
16	Amino acid	L-Proline	13.384	$C_5H_9NO_2$	0.891
17	Amino acid	Glycine	13.567	$C_2H_5NO_2$	1.154
18	Amino acid	Serine	14.551	$C_3H_7NO_3$	0.866
19	Amino acid	L-Threonine	14.997	$C_4H_9NO_3$	1.027
20	Amino acid	5-Oxoproline	17.074	$C_5H_7NO_3$	1.138
21	Amino acid	L-Glutamic acid	18.533	$C_5H_9NO_4$	1.330
22	Amino acid	L-Phenylalanine	22.779	$C_9H_{11}NO_2$	0.800
23	Amino acid	Creatinine	17.595	$C_4H_7NO_3$	1.025
24	Polyol	Myo-inositol	24.765	$C_6H_{12}O_6$	1.351
25	Other	Cholesterol	34.715	$C_{27}H_{46}O$	0.915
26	Other	n-Butylamine	16.073	$C_4H_{11}N$	0.675
27	Other	Eicosyl isopropyl ether	29.354	$C_{23}H_{48}O$	1.119
28	Other	1-Monopalmitin	29.680	$C_{19}H_{38}O_4$	0.561
29	Other	Glycerol monostearate	31.437	$C_{21}H_{42}O_4$	0.909
30	Other	3-Cyclohexene-1-ethanol	34.944	$C_8H_{14}O$	0.852
31	Other	Propyleneglycol monoleate	36.729	$C_{21}H_{40}O_3$	1.382
32	Other	1-naphthalenamine	37.788	$C_{10}H_9N$	0.817

Supplemental Table S3. Identification of extracellular metabolites by GC-MS.

No.	Class	Metabolite identity	Retention time	Molecular formula	VIP
1	Sugar	D-Fructose	22.173	$C_6H_{12}O_6$	1.052
2	Sugar	D-Galactose	22.722	$C_6H_{12}O_6$	0.805
3	Sugar	D-Mannose	22.499	$C_6H_{12}O_6$	1.138
4	Organic acid	1,2,3-Propanetricarboxylic acid	21.372	$C_6H_8O_6$	1.090
5	Organic acid	2,3,4-Trihydroxybutyric acid	17.544	$C_4H_8O_5$	0.891
6	Organic acid	2-Piperidinecarboxylic acid	17.051	$C_6H_{11}NO_2$	1.040
7	Fatty acid	Hexadecanoic acid	23.895	$C_{16}H_{32}O_2$	1.039
8	Fatty acid	Octadecanoic acid	26.115	$C_{18}H_{36}O_2$	0.780
9	Amino acid	L-Ornithine	21.263	$C_5H_{12}N_2O_2$	0.859
10	Amino acid	Glycine	13.561	$C_2H_5NO_2$	1.018
11	Amino acid	Alanine	9.865	$C_3H_7NO_2$	0.887
12	Amino acid	L-Asparagine	19.283	$C_4H_8N_2O_3$	1.066
13	Amino acid	L-Aspartic acid	16.336	$C_4H_7NO_4$	1.096
14	Amino acid	L-Cysteine	17.589	$C_3H_7NO_2S$	1.117
15	Amino acid	L-Leucine	12.977	$C_6H_{13}NO_2$	0.939
16	Amino acid	L-Lysine	22.562	$C_6H_{14}N_2O_2$	1.011
17	Amino acid	L-Phenylalanine	18.596	$C_9H_{11}NO_2$	1.114
18	Amino acid	L-Proline	13.378	$C_5H_9NO_2$	1.030
19	Amino acid	L-Threonine	14.992	$C_4H_9NO_3$	0.773
20	Amino acid	L-Tyrosine	22.785	$C_9H_{11}NO_3$	0.877
21	Amino acid	L-Valine	11.976	$C_5H_{11}NO_2$	1.120
22	Polyol	Arabitol	20.130	$C_5H_{12}O_5$	1.012
23	Polyol	D-Mannitol	22.991	$C_6H_{14}O_6$	0.970
24	Polyol	Myo-inositol	24.770	$C_6H_{12}O_6$	0.912
25	Polyol	Ribitol	22.304	$C_5H_{12}O_5$	1.105
26	Other	3-Methyl-3-pentanol	22.007	$C_6H_{14}O$	0.870
27	Other	Acetamide	20.862	C_2H_5NO	1.137
28	Other	Butane	16.994	C_4H_{10}	1.031
29	Other	Glutamine	18.528	$C_5H_{10}N_2O_3$	1.103
30	Other	n-Butylamine	16.062	$C_4H_{11}N$	0.661
31	Other	Urea	12.371	CH_4N_2O	1.085
